# 942. Improving Accuracy in Documentation and Data Abstraction with a Burn Tertiary Survey Note

**DOI:** 10.1093/jbcr/irag033.509

**Published:** 2026-04-06

**Authors:** Leslie A Miller, Carey K Lamphier, Jasmin Mercedes, Debbie Boyett, Kristen Glasgow-Petela, Laura S Johnson, Lauren B Nosanov

**Affiliations:** Grady Memorial Hospital, Atlanta, GA; Grady Memorial Hospital, Atlanta, GA; Grady Memorial Hospital, Atlanta, GA; Grady Memorial Hospital, Atlanta, GA; Morehouse School of Medicine, Decatur, GA; Grady Memorial Hospital / Emory School of Medicine / Morehouse School of Medicine, Atlanta, GA; Emory University School of Medicine, Atlanta, GA

## Abstract

**Introduction:**

Uncertainty is an inherent component of caring for critically ill and traumatically injured patients. Often a patient’s identity is unknown; incomplete information creates challenges in patient care and quality improvement (QI). A Burn Service tertiary survey note was modeled after existing institutional Trauma documentation to improve the accuracy and completeness of patient information.

**Methods:**

Patterns of inaccurate data elements from the History and Physical (H&P) were identified. A dot phrase was created, incorporating standard elements from the trauma tertiary note and unique burn-specific elements. Following education with clinicians, the process went live in July 2024. Patients admitted to the Burn service were audited. Note completion was prospectively tracked and note content form was adjusted based on challenges and feedback. In May 2025 a retrospective assessment was performed to identify trends in completion compliance. In addition, QI team members’ feedback was qualitatively analyzed to identify themes and opportunities for improvement.

**Results:**

A total of 542 patients managed by the burn service were reviewed; 40 comanaged patients, 14 patients admitted less than 24 hours, and 20 patients with incomplete data were excluded, leaving 468 patients. Overall compliance with note completion was 35.9%. Note completion tended to be higher amongst service Advanced Practice Providers in comparison with rotating trainees. The QI team found the burn tertiary note to be equivalently reliable as a source for data abstraction as the H&P. In addition, the QI team noted the quality of the burn tertiary note to be user dependent. The evaluation of the efficacy of the Burn tertiary note is ongoing.

**Conclusions:**

The implementation of the Burn tertiary note has addressed some of the challenges associated with incomplete patient information. We continue to address the identified human factors impacting compliance, thoroughness, and accuracy. Focus groups are planned with attendings, APPs, and the Trauma QI program to identify next steps. We aim for conversion of the tertiary note to an electronic medical record form creating discrete data elements for facile extraction and registry completion.

**Applicability of Research to Practice:**

A Burn tertiary note provides a reliable process for complete and accurate patient information to be entered into the patient’s chart in the event the information is not available at time of admission. Complete and accurate patient information is vital to achieving best possible patient outcomes and supports a through QI process.

**Funding for the study:**

N/A.

